# A Fast and Accurate Sparse Continuous Signal Reconstruction by Homotopy DCD with Non-Convex Regularization

**DOI:** 10.3390/s140405929

**Published:** 2014-03-26

**Authors:** Tianyun Wang, Xinfei Lu, Xiaofei Yu, Zhendong Xi, Weidong Chen

**Affiliations:** 1 Key Laboratory of Electromagnetic Space Information, Chinese Academy of Sciences, University of Science and Technology of China, Hefei 230027, China; E-Mails: wangty@mail.ustc.edu.cn (T.W.); lxfei@mail.ustc.edu.cn (X.L.); yuxfagn@mail.ustc.edu.cn (X.Y.); 2 China Satellite Maritime Tracking and Control Department, Jiangyin 214431, China; E-Mail: zhengdongxi1@gmail.com

**Keywords:** compressed sensing, sparse continuous signal recovery, off-grid problem, low-complexity reconstruction, non-convex regularization

## Abstract

In recent years, various applications regarding sparse continuous signal recovery such as source localization, radar imaging, communication channel estimation, *etc.*, have been addressed from the perspective of compressive sensing (CS) theory. However, there are two major defects that need to be tackled when considering any practical utilization. The first issue is off-grid problem caused by the basis mismatch between arbitrary located unknowns and the pre-specified dictionary, which would make conventional CS reconstruction methods degrade considerably. The second important issue is the urgent demand for low-complexity algorithms, especially when faced with the requirement of real-time implementation. In this paper, to deal with these two problems, we have presented three fast and accurate sparse reconstruction algorithms, termed as HR-DCD, Hlog-DCD and H*l_p_*-DCD, which are based on homotopy, dichotomous coordinate descent (DCD) iterations and non-convex regularizations, by combining with the grid refinement technique. Experimental results are provided to demonstrate the effectiveness of the proposed algorithms and related analysis.

## 1. Introduction

Compressive sensing (CS) and sparse signal representation have received widespread attention and increasing interest over the past few years [1,2], which are motivated by the sparse nature of the real world data and the advantages of the CS theory. The applications of CS in numerous areas have been widely investigated in the literature, such as magnetic resonance imaging (MRI) [3], synthetic aperture radar (SAR) imaging [4], inverse synthetic aperture radar (ISAR) imaging [5], passive radar imaging [6], direction-of-arrival (DOA) estimation [7], communication channel estimation [8], seismic signal processing [9], spectral estimation [10], image processing [11], speech enhancement [12], *etc.* Generally speaking, those disciplines explore the following linear signal model [13]:

(1) y=Hw+n

where **y** ∈ ℂ^***M***×**1**^ is a *M* × 1complex vector, **H** ∈ ℂ*^**M**^*^×^*^**N**^* is a *M* × *N* complex measurement matrix, **w** ∈ ℂ*^N^*^×^**^1^** represents the *N* × 1 unknown complex signals of interest, and **n** ∈ ℂ*^M^*^×^**^1^** denotes a *M* × 1complex noise vector. Suppose **w** is sparse or compressible in a known dictionary **Ψ** ∈ ℂ*^**N**^*^×^*^**N**^*, *i.e.*, **w** = **Ψx**, where **x** ∈ ℂ*^N^*^×^**^1^** is a *K*-sparse vector, namely it can be approximated by its *K* largest coefficients or its coefficients satisfy a power decay law with *K* strongest coefficients. Therefore, linear measurements are obtained in CS as:

(2) y=Ax+n

where **A = HΨ** ∈ ℂ*^**M**^*^×^*^**N**^* represents the sensing matrix. Although the above equation is usually ill-posed, the CS theory has shown that if **A** satisfies some certain conditions, we can construct **x** and **w** stably from highly undersampled measurements **y** [14].

### 1.1. Off-Grid Problem in CS-Based Methods

In the CS processing procedure, the first necessary step is to design a dictionary through a discretization operation with the assumption that the elements of unknown **x** lie exactly on those pre-defined grids corresponding to **A**. Obviously, this is practically impossible since the continuous nature of the unknowns of **x**, such as the unknown directions, may not fall into the predefined angular grids in DOA, and the scatterers of the target may not locate exactly on the pre-discretized scene grids in radar imaging. Hence, once faced with a continuous signal, the off-grid problem in using the conventional sparse recovery techniques is inevitable, no matter how densely we grid **x**. Previous researches [15–17] have demonstrated that the traditional CS-based methods would be severely affected when the off-grid problem emerges, and [18] also claimed that the off-grid problem is one of the major constraints in popularizing CS-based methods in practice. It is worth noting that there may be other factors leading to basis mismatch, for example, in the radar imaging field, unsatisfactory system parameters (e.g., position errors of transceivers [19] and phase synchronization mismatch [20]) are also likely to degrade the performance of conventional CS-based methods from our previous researches, however this paper only focuses on the off-grid problem, and assumes the system errors are small enough.

The solutions for off-grid problem have been broadly studied in previous literatures [21–30]. So far, main research topics include three types as follows:

(a) *Direct method according to theories of Xampling and the finite rate of innovation (FRI)*. This scheme is first introduced by Michaeli *et al.* as a general framework within various solutions for analog signals [21]. The significant advantage of this method is its direct solution, without any pre-discretization of the continuous signal at first. Thus it is less sensitive to off-grid problem compared with traditional CS-based methods. However, this method is mainly based on spectral estimation algorithms (e.g., ESPIRT [22], MUSIC [6], Matrix Pencil [23]), which needs special signal expression in linear measurement model and its performance may suffer from low SNR and few snapshots [24].
(b) *Off-grid CS method*. This kind of method is under a first order Taylor expansion model, and thus is quite sensitively depended on the model's accuracy, and cannot give a thorough solution when higher order approximations are significant [23]. Furthermore, it is likely to involve highly-computational burden [25,26] for alternatively finding the sparse solution and estimating the off-grid error, especially in large scale problem.
(c) *Grid refinement approach*. The idea of the grid refinement technique was firstly introduced by Malioutov *et al.* [27] to mitigate the effect of limiting estimates to a grid of spatial locations in source localization problem. Then it is generalized by Liu *et al.* [28] for locating two-dimensional multiple underwater acoustic sources. Recently, Guldogan *et al.* [29] have proposed a novel grid refinement algorithm to alleviate off-grid problem by using a particle swarm optimization (PSO) and orthogonal matching pursuit (OMP), which makes use of the PSO to perturb the location of each grid point.

Since the “grid refinement approach” is iteratively refined to match with the desired resolution of the off-grid components by “coarse and fine grid partition” operations, and does not have the drawbacks of (a) and (b), this paper follows the idea of “grid refinement approach” which makes a coarse grid first instead of having an universally fine grid to reduce the complexity, then achieves fine grids around the peaks using more refinement levels. In this way, after a few iterations of refining process, it becomes fine enough that off-grid problem effect is negligible.

### 1.2. Fast and Efficient Algorithms for Real-Time Implementation

Furthermore, there exists another common challenge by utilizing the CS-based methods, *i.e.*, we need fast and efficient algorithms for real-time system implementation, particularly for digital electronic circuits (e.g., ARM, FPGA, DSP) [30]. From previous researches, sparse recovery techniques can be roughly divided into two families, that are greedy methods (e.g., MP [31], OMP [32], GP [33]) and optimization based methods (e.g., *l*_1_ optimization [34], smoothed *l*_0_ optimization [35], non-convex optimization [36]). Generally speaking, greedy methods have the advantages of lower complexity, faster speed, less storage requirement, and flexible implementation compared with optimization based methods, and are considered the most suitable candidates for hardware implementation. However, their performances are inferior to those of the optimization based methods, such as *l*_1_ norm minimization basis pursuit de-noising (BPDN) [34].

As the coordinate descent (CD) search has an inherent property of being low complexity when signals are sparse, Zakharov *et al.* have successfully exploited dichotomous CD (DCD) iterations for solving LS [37], RLS [38] and MVDR beamforming problems [39] using FPGAs for real-time implementation. In addition, to solve the reweighted *l*_1_ minimization problem, they have developed a greedy algorithm called H*l*1-DCD [40], *i.e.*, homotopy DCD method with reweighted *l*_1_ penalty, which has shown a high recovery performance with relatively low complexity. It turns out that the overall complexity of the algorithm is comparable to that of MP. Moreover, homotopy iterations result in high accuracy of the solution, even higher than that of the YALLl algorithm. Meanwhile, Liu and Zakharov *et al.* [28] have utilized the low complexity homotopy approach combined with CD search for locating underwater acoustic sources by solving the multi-frequency BPDN problem. The proposed method is evaluated by applying to simulated and real experimental data. As far as we know, previous studies [28,37–40] are mainly focusing on homotopy DCD with convex regularizations, while there are seldom researches linking to homotopy DCD with non-convex regularizations.

### 1.3. Our Contribution

From the above discussion, we have addressed two major issues (*i.e.*, off-grid problem and efficient algorithms for real-time implementation) of applying CS to sparse continuous signal reconstruction. In this paper, motivated by the idea of H*l*1-DCD [40] and grid refinement approach [27], we present three fast and accurate sparse reconstruction algorithms (*i.e.*, HR-DCD, Hlog-DCD and H*l_p_*-DCD) which are based on homotopy, dichotomous coordinate descent (DCD) iterations and non-convex regularization, combining with the grid refinement technique to deal with the aforementioned issues. The main contributions of this paper are as follows: (1) we formulate the sparse recovery problem by homotopy DCD method with three typical classes of non-convex penalties, which are proved to recover sparsity in a more efficient way than homotopy DCD method with convex penalties as shown in Zakharov's previous researches [28,37–40]; (2) further, the grid refinement technique [27] is utilized to combine with our algorithms to alleviate the effect of off-grid problem and reduce the complexity simultaneously; (3) experimental results of three representative applications (DOA, passive radar imaging, ISAR imaging) are carried out to verify the effectiveness of the proposed methods.

The outline of this paper is as follows: in Section 2, two sparse recovery problems are discussed. Section 3 describes the proposed methods and related analysis. In Section 4, extensive experimental results are presented to verify our methods. Finally, Section 5 draws the conclusions.

Throughout this paper we shall make use of the following notation: bold-case letters are reserved for vectors and matrices, e.g., **x** is a vector, **A** is a matrix; Elements of **A** and **x** are represented as A*_n,p_* and x*_n_*, respectively. *I* and *I^c^* denote the support and its complement, respectively. Further, we represent by **R**^(^*^q^*^)^ the *q*-th column of **R; A***_I_* a matrix obtained from **A** keeping only columns corresponding to *I*; **x***_I_* the subset of **x** that contains entries from **x** corresponding to *I*; ‖**x**‖*_p_* denotes the *l_p_* norm of a vector, ‖**A**‖_2_ is the spectral norm of the matrix **A**; 〈,〉 denotes the inner product; (·)^H^, (·)*, Re{·}denote the conjugate transpose, conjugate and real part operations, respectively.

## 2. Problem Formulation and Motivations

This section briefly introduces the general sparse recovery framework, upon which we develop our algorithms. As stated before, various applications can be represented by the linear model as Equation (2) shows.

**Figure 1.**
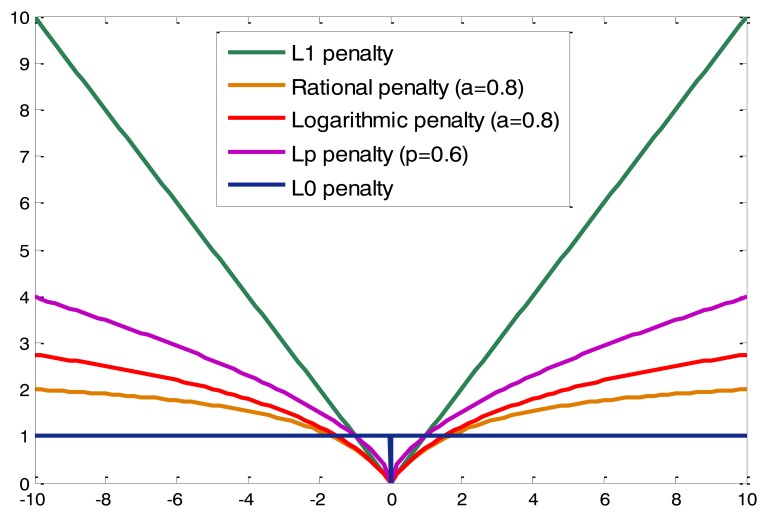
Examples of different penalty functions.

When considering the practical applications, sparse recovery techniques require low-complexity algorithms just as some kinds of greedy methods which are suitable for real-time implementation. However, the performances of greedy methods are compared unfavorably with optimization based methods (e.g., using *l*_1_ norm, reweighted *l*_1_ norm, *l_p_* norm, smoothed *l*_0_ regularization). Recently, Zakharov *et al.* have proposed the homotopy DCD method with convex regularizations, including *l*_1_ and reweighted *l*_1_ norms, which have shown a high recovery performance and relatively low complexity. Moreover, most of the operations in the algorithms are additions, therefore, they are very suited for the hardware implementation of real-time operating systems. Motivated by the idea of homotopy DCD method and the fact that non-convex regularization usually yields a sparser solution than any convex penalty for a given residual energy (see Figure 1 for example), this paper proposes a fast and accurate sparse signal reconstruction by homotopy DCD technique with non-convex regularizations. Three typical non-convex penalty functions [12,41] are considered, *i.e.*, the first order rational function penalty, the logarithmic penalty and the *l_p_* penalty, respectively. With the corresponding penalties, we have achieved the derivation of the HR-DCD, Hlog-DCD and H*l_p_*-DCD algorithms in the next section.

**Figure 2.**
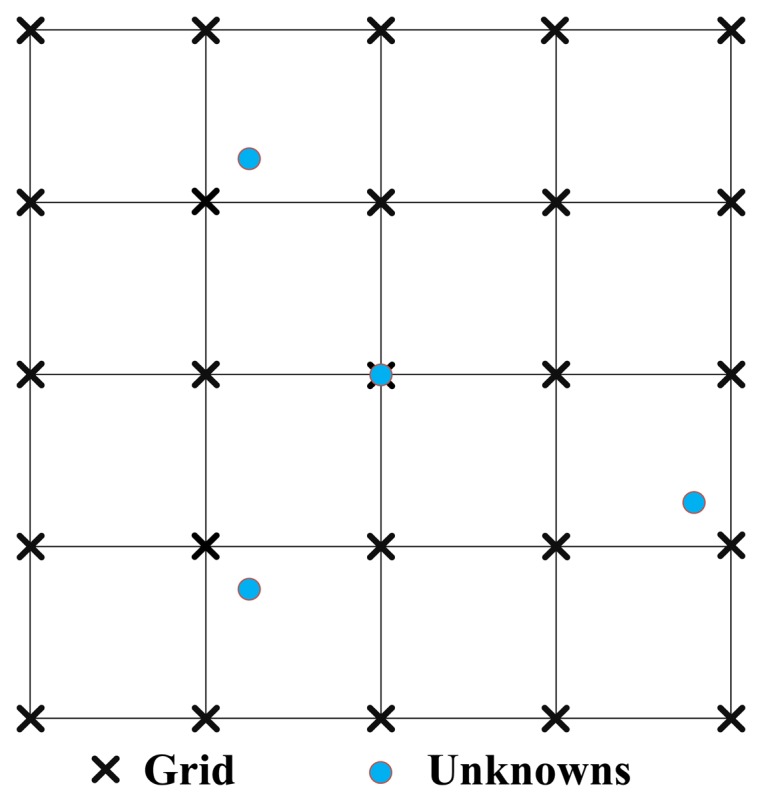
Off grid and on grid scatterers.

**Figure 3.**
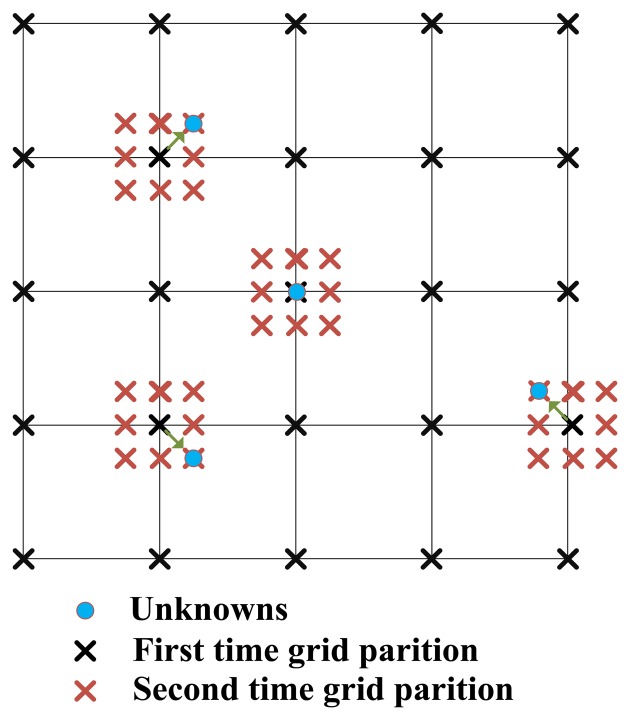
Illustration of grid refinement approach.

Since **x** is usually distributed continuously in the corresponding space in many applications, the off-grid problem is likely to exist. For example, in the radar imaging field, the reflectivity centers of the scatterers are generally not located at exact on-grid spatial positions illustrated in Figure 2, which means the measurement matrix should cover the basis vectors corresponding to off-grid scatterers. As conventional CS-based methods do not consider the off-grid effect, therefore the related sparse reconstruction techniques suffer unacceptable degradation in image quality. This paper has utilized the grid refinement approach [27] shown in Figure 3 combined with the HR-DCD, Hlog-DCD and H*l_p_*-DCD algorithms to alleviate the impact of off-grid problem (see details in Section 3.2). Experimental results in Section 4 are provided to demonstrate the performance improvement of the proposed algorithms.

## 3. Homotopy DCD with Non-Convex Regularization Algorithms

We consider minimization of the following cost function to solve the problem Equation (2):

(1) $$J\left[x\right]=\frac{1}{2}{\left\|y-Ax\right\|}_{2}^{2}+\tau \rho \left(x\right)$$

where the sparsity measurement function *ρ*(**x**) is generally separable, or *ρ*(**x**)= Σ*_i_ φ* (x*_i_*) where *φ*(·) denotes the parameterized form and satisfies such conditions that *ρ*(**x**) is sparsity-preserving, and τ ≥ 0 is a regularization parameter. As mentioned in previous researches, we can use DCD iterations for minimizing the cost function, which is represented in a fixed point. The DCD algorithm is appealing for practical designs as it operates at the bit level, resulting in stable hardware implementations, which is shown in Table 1. Different from Zakharov's studies [28,37–40], this paper utilizes the non-convex regularizations instead of convex regularizations embedding with the DCD algorithm, and chooses three typical penalty functions as follows:

(a) The first order rational function penalty:
  (2) $$\phi \left(x_{i}\right)=\frac{\left|x_{i}\right|}{1+\frac{a}{2}\left|x_{i}\right|}$$
(b) The logarithmic penalty:
  (3) $$\phi \left(x_{i}\right)=\frac{1}{a}\log\left(1+a\left|x_{i}\right|\right)$$
(c) The *l_p_* penalty:
  (4) $$\phi \left(x_{i}\right)={\left|x_{i}\right|}^{p}$$

In order to reduce the complexity of the DCD algorithm, we develop a greedy algorithm that is based on homotopy method with respect to a set of the parameter *τ*. Besides, the updates of our DCD algorithm are only done within the support instead of all elements. As the support is usually much smaller than *N*, therefore, the complexity is further reduced. We consider the first order rational function penalty at first, and develop HR-DCD algorithm. Then Hlog-DCD algorithm and H*l_p_*-DCD algorithm can be similarly obtained.

The following two propositions define the rules for adding/removing elements into/from the support of HR-DCD algorithm.

Let *I* be the support at some homotopy iteration, and *I^c^* be its complementary set. Denote **r = y − Ax**, **R = A***^H^* **A** and **b = A***^H^* **r**.

**Proposition 1**: Add the *t*-th (*t* ∈ *I^c^*) element into the support *I* according to the rule:

(5) $$\begin{array}{cc} t=\arg\underset{k\in I^{c}}{\max}\frac{a\left|b_{k}\right|-R_{k,k}}{aR_{k,k}^{2}}\left\{{\left(R_{k,k}+a\left|b_{k}\right|\right)}^{2}-4aR_{k,k}\tau \right\} & s.t.{\left(R_{t,t}+a\left|b_{t}\right|\right)}^{2}>4aR_{t,t} \end{array}\tau$$

**Proposition 2**: Remove the *t*-th (*t* ∈ *I*) element from the support *I* according to the rule

(6) $$\begin{array}{cc} t=\arg\underset{k\in I}{\min}\frac{1}{2}{\left|x_{k}\right|}^{2}R_{k,k}+ℜ\left\{x_{k}^{*}b_{k}\right\}-\tau \frac{\left|x_{k}\right|}{1+\frac{a}{2}\left|x_{k}\right|} & s.t.\frac{1}{2}{\left|x_{t}\right|}^{2}R_{t,t}+ℜ\left\{x_{t}^{*}b_{t}\right\}-\tau \frac{\left|x_{t}\right|}{1+\frac{a}{2}\left|x_{t}\right|} \end{array}<0$$

We now prove Proposition 1.

***Proof***: Let the *t*-th element *x_t_* = 0 be activated as *x̂* = *α* = |*α*|*e ^j^*^arg{α}^, and the updated solution vector is denoted as **x̂** The update of the cost function Equation (3) is then given by:

(7) $$\Delta J=J\left[\hat{x}\right]-J\left[x\right]=\frac{1}{2}{\left|\alpha \right|}^{2}R_{t,t}-ℜ\left\{\alpha^{*}b_{t}\right\}+\tau \frac{\left|\alpha \right|}{1+\frac{a}{2}\left|\alpha \right|}$$

The cost function is reduced if Δ*J* < 0. For a fixed |*α*|, Δ*J* achieves a minimum value if arg{*α*} = arg{b*_t_*}, and in this case:

(8) $$\Delta J=\frac{1}{2}{\left|\alpha \right|}^{2}R_{t,t}-\left|\alpha \right|\left|b_{t}\right|+\tau \frac{\left|\alpha \right|}{1+\frac{a}{2}\left|\alpha \right|}$$

Let:

(9) $$\begin{array}{ll} \Delta J\prime & =1\left(1+\frac{a}{2}\left|\alpha \right|\right)\Delta J\\ & =\left|\alpha \right|\left\{aR_{t,t}{\left|\alpha \right|}^{2}\left(R_{t,t}-a\left|b_{t}\right|\right)\left|\alpha \right|+4\left(\tau -\left|b_{t}\right|\right)\right\}\\ & =aR_{t,t}\left|\alpha \right|\left\{{\left(\left|\alpha \right|+\frac{R_{t,t}-a\left|b_{t}\right|}{aR_{t,t}}\right)}^{2}-{\left(\frac{R_{t,t}-a\left|b_{t}\right|}{aR_{t,t}}\right)}^{2}+\frac{1\left(\tau -\left|b_{t}\right|\right)}{aR_{t,t}}\right\} \end{array}$$

For |*α*| > 0, if $\left|\alpha \right|=-\frac{R_{t,t}-a\left|b_{t}\right|}{aR_{t,t}}$, we have:

(10) $$\Delta J\prime =\frac{1}{aR_{t,t}}\left|\alpha \right|\left\{4aR_{t,t}\tau -{\left(R_{t,t}+a\left|b_{t}\right|\right)}^{2}\right\}$$

If Δ*J*′ < 0, then 4*aR_t,t_ τ* − (*R_t,t_* + *a*|*b_t_*|)^2^ < 0. Thus, there exists a value of *α* that results in Δ*J* < 0, *i.e.*, in reducing the cost function. It is seen from the last expression that if we want to add a new element to the solution vector, the index *t* of the element should correspond to the maximum of $\frac{a\left|b_{t}\right|-R_{t,t}}{aR_{t,t}^{2}}\left\{{\left(R_{t,t}+a\left|b_{t}\right|\right)}^{2}-4aR_{t,t}\tau \right\}$ over *t* ∈ *I^c^*. In this case, we will obtain the largest decrement of the cost function.

Thus, the value of *α* that for a fixed *t* such that 4*aR_t,t_ τ* − (*R_t,t_* + *a*|*b_t_*|)^2^ < 0 results in the decrement of the cost function is given by:

(11) $$\alpha =\frac{a\left|b_{t}\right|-R_{t,t}}{aR_{t,t}}e^{j\arg\left\{b_{t}\right\}}$$

According to the above statements, the (*n* + 1)-th nonzero element to be activated in **x̂** should satisfy:

(12) $$\begin{array}{cc} t=\arg\underset{k\in I^{c}}{\max}\frac{a\left|b_{k}\right|-R_{k,k}}{aR_{k,k}^{2}}\left\{{\left(R_{k,k}+a\left|b_{k}\right|\right)}^{2}-4aR_{k,k}\tau \right\} & s.t.{\left(R_{t,t}+a\left|b_{t}\right|\right)}^{2}>4aR_{t,t}\tau \end{array}$$

For |*α*| > 0, we have:

(13) $$\begin{array}{ll} \Delta J & \geq \frac{1}{2}{\left|\alpha \right|}^{2}R_{t,t}-\left|\alpha \right|\left|b_{t}\right|+\tau \frac{\left|\alpha \right|}{1+\frac{a}{2}\left|\alpha \right|}\\ & \geq \frac{\left|\alpha \right|}{4\left(1+\frac{a}{2}\left|\alpha \right|\right)}\left\{\left(\left|\alpha \right|R_{t,t}-2\left|b_{t}\right|\right)\left(2+a\left|\alpha \right|\right)+4\tau \right\} \end{array}$$

If (*R_t,t_* + *a*|*b_t_*|)^2^ < 4*aR_t,t_ τ*, then in this case:

(14) $$\begin{array}{ll} \Delta J & >\frac{\left|\alpha \right|}{4\left(1+\frac{a}{2}\left|\alpha \right|\right)}\left\{\left(\left|\alpha \right|R_{t,t}-2\left|b_{t}\right|\right)\left(2+a\left|\alpha \right|\right)+\frac{1}{aR_{t,t}}{\left(R_{t,t}+a\left|b_{t}\right|\right)}^{2}\right\}\\ & >\frac{\left|\alpha \right|}{4aR_{t,t}\left(1+\frac{a}{2}\left|\alpha \right|\right)}{\left(aR_{t,t}\left|\alpha \right|+R_{t,t}-a\left|b_{t}\right|\right)}^{2}\geq 0 \end{array}$$

Therefore, if |*α*|> 0 then for (*R_t,t_* + *a*|*b_t_*|)^2^ < 4*aR_t,t_ τ*, we have Δ*J* > 0, *i.e.*, the cost function increases.

We now prove the Proposition 2:

***Proof***: Let the *t*-th element *x_t_* ≠ 0 be activated as *x̂_t_* = 0 and the updated solution vector is denoted as **x̂**. The update of the cost function is then given by:

(15) $$\Delta J=J\left[\hat{x}\right]-J\left[x\right]=\frac{1}{2}{\left|x_{t}\right|}^{2}R_{t,t}+ℜ\left\{x_{t}^{*}b_{t}\right\}-\tau \frac{\left|x_{t}\right|}{1+\frac{a}{2}\left|x_{t}\right|}$$

Thus, if |xt|2Rt,t/2+Re{xt*bt}−τ|xt|/(1+a2|xt|)<0, then Δ*J* < 0, there exists a nonzero value of the *t*-th element that decreases the cost function that should be removed from the support.

Combining the DCD algorithm in Table 1 and the above two propositions, we arrive at the HR-DCD algorithm presented in Table 2.

The HR-DCD algorithm starts with the zero support *I* = Ø, and *τ* = |*b_t_*|. For each *τ*, it updates the support and minimizes the cost function according to the corresponding propositions. Besides, the algorithm will terminate if the iteration times reach the preset parameter *L* or *τ* ≤ *τ*_min_ (*τ*_min_ is a predefined parameter).

**Remark 1:** When the penalty is logarithmic function, we give two similar propositions as follows:

**Proposition 3:** Add the *t*-th (*t* ∈ *I^c^*) element into the support according to the rule:

(16) $$\begin{array}{cc} t=\arg\underset{k\in I^{c}}{\max}\left|b_{k}\right| & s.t.\left|b_{t}\right|>\tau \end{array}$$

**Proposition 4:** Remove the *t*-th (*t* ∈ *I*) element from the support according to the rule:

(17) $$\begin{array}{cc} t=\arg\underset{k\in I}{\min}\frac{1}{2}{\left|x_{k}\right|}^{2}R_{k,k}ℜ\left\{x_{k}^{*}b_{k}\right\}-\tau \frac{1}{a}\log\left(1+a\left|x_{k}\right|\right) & s.t.\frac{1}{2}{\left|x_{t}\right|}^{2}R_{t,t}ℜ\left\{x_{t}^{*}b_{t}\right\}-\tau \frac{1}{a}\log\left(1+a\left|x_{t}\right|\right) \end{array}<0$$

**Remark 2:** When the penalty is *l_p_* norm, we can also give two similar propositions as follows:

**Proposition 5:** Add the *t*-th (*t* ∈ *I^c^*) element into the support according to the rule:

(18) $$\begin{array}{cc} t=\arg\underset{k\in I^{c}}{\max}\left|b_{k}\right| & s.t.{\left|b_{t}\right|}^{2-p}>2\tau R_{t,t}^{1-p} \end{array}$$

**Proposition 6:** Remove the *t*-th (*t* ∈ *I*) element from the support according to the rule:

(19) $$\begin{array}{cc} t=\arg\underset{k\in I}{\min}\frac{1}{2}{\left|x_{k}\right|}^{2}R_{k,k}+ℜ\left\{x_{k}^{*}b_{k}\right\}-\tau {\left|x_{k}\right|}^{p} & s.t. \end{array}\frac{1}{2}{\left|x_{t}\right|}^{2}R_{t,t}+ℜ\left\{x_{t}^{*}b_{t}\right\}-\tau {\left|x_{t}\right|}^{p}<0$$

The proofs for Equations (18)–(21) will be shown in the Appendix. By combining the corresponding propositions with related DCD algorithms, it is easy to obtain Hlog-DCD algorithm and H*l_p_*-DCD algorithm, respectively, and we omit the tabulated expressions here for simplicity.

### 3.1. Complexity Analysis

The complexity is given by *P* ≅ 8*MN* + 4*NL* + 2*C_u_N* +*C_i_* +2*N_deb_L_g_* The term 8*MN* is for computing the initial **b**. The term 4*NL* is for selection of elements in the support. The term 2*C_u_N* is for updating **b** in the total number *C_u_* of successful DCD iterations, and each update requires only 2*N* real valued additions, as multiplications by *α_t_* are bit-shifts. The term *C_i_* takes into account *C_i_* (total number of DCD iterations) tests to decide if the DCD iteration is successful. The debiasing (2*N_deb_L_g_*) is now done by using extra *N_deb_* DCD iterations on the finally identified support with size *L_g_*.

### 3.2. Off-Grid Problem Solution

As stated before, we explore the idea of grid refinement technique [27] to reduce the number of the arbitrarily located potential positions of **x**. Two-dimensional passive radar imaging is selected as an example to illustrate the procedures of solution, which is realized as follows:

(1) Under a coarse resolution Δ*x_q_*, Δ*y_q_*, where Δ*x_q_*, Δ*y_q_* represent the range step and azimuth step respectively, calculate the coarse localizations (*x_n_*, *y_n_*), = 1, ⋯, *S* results by finding *S* largest amplitude peaks using the proposed algorithms, *i.e.*, HR-DCD, Hlog-DCD and H*l_p_*-DCD.
(2) Build a denser grid around the estimated locations (*x_n_*, *y_n_*) with a finer resolution (Δ*x_q_*, Δ*y_q_*) as Figure 2 mentioned in Section 2.
(3) Set *q* = *q* + 1 and $\Delta x_{q}=\frac{1}{2}\Delta x_{q}$, $\Delta y_{q}=\frac{1}{2}\Delta y_{q}$, repeat for *Q* times from Step (1) until the desired resolution is achieved.

In order to reduce the multiplications, we use dichotomy for grid refinement, which can use bit-shifts instead of multiplications to be implement in hardware system. According to our experimental results in Section 4, no more than 10-step grid refinement process is needed.

### 3.3. Extension to Multiple Measurement Vectors (MMV) Case

Although we have derived HR-DCD, Hlog-DCD and H*l_p_*-DCD algorithms from a single measurement vector (SMV), the results can be generalized to MMV case. In some kinds of applications, such as wideband source localization [42], multiple frequency bins are explored. As we all know that single frequency results can be easily disturbed by noise and interference, and in this issue, we can use a frequency diversity to achieve better performance. A traditional method of modifying the above approach to multi-frequency signals is averaging the results of all the frequencies. But due to the presence of noise, environmental mismatch and interference, different frequencies may give different results, thus this combining method may not work well.

By the similar approach used in [28], our proposed methods require that for all frequencies to have the same support, which utilize the joint sparsity pattern, and choose an element to add to the support according to the corresponding propositions to achieve greater frequency diversity and avoid the possible wrong results. And the final result can be given by averaging all the results of all the frequencies through this way.

## 4. Experimental Results

In this section, we will present several simulation results which illustrate the effectiveness of the proposed methods. All experiments are performed by using MATLAB R2013a on a PC equipped with an Inter Core i7 3770k CPU, 3.5GHz and 32 GB memory. The state-of-the-art sparse recovery methods such as MP, BP, FOCUSS, SBL, H*l*1-DCD, *etc.* are selected for comparison. As a benchmark we will utilize an oracle sparse recovery (OSR) to represent the best inversion performance, which knows the true support of **x**. In addition, we set parameter *a* = 10 in Equation (4) for HR-DCD, in Equation (5) for Hlog-DCD, and *p* = 0.8 in Equation (6) for H*l_p_*-DCD, and *M_b_* = 10, *τ*_min_ = 0.02, *γ* = 0.9 by experience from extensive simulation results. Other predefined parameters such as *H*, *L*, *S*, and the grid refinement times *Q*, *etc.*, are decided according to the actual conditions, which will be given in details in the next text.

### 4.1. DOA Estimation

**Figure 4.**
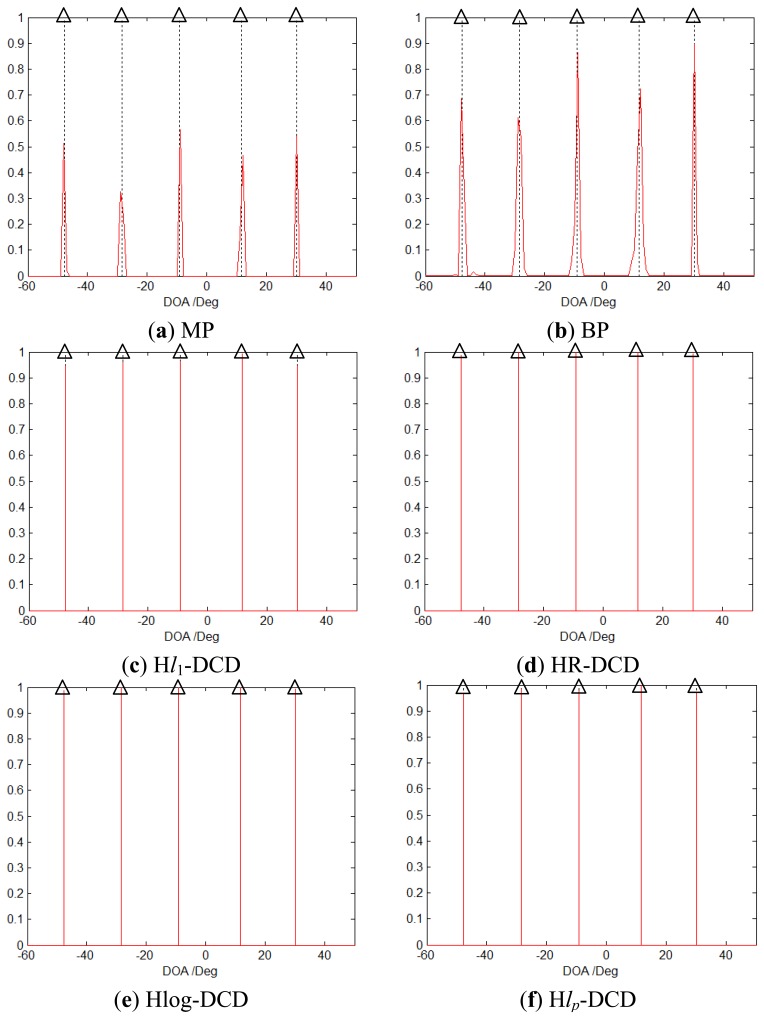
Solutions of MP (**a**), BP (**b**), H*l*_1_-DCD (**c**), HR-DCD (**d**), Hlog-DCD (**e**), H*l_p_*-DCD (**f**) for off-grid DOAs. True DOAs are denoted by the vertical dashed lines.

As described by Malioutov *et al.*, for one snapshot case, the DOA problem of Equation (5) in [27] is equivalent to Equation (2) in this paper. With the assumption that the sources can be viewed as point sources and their number is small, the underling spatial spectrum is sparse and it can be solved via sparse recovery methods mentioned above. The first simulation considers the scenario that there are five uncorrelated signals impinging from [−47.7°, −28.5°, −9.2°, 11.6°, 30.1°]. The grid is set to be within the range of −90° to 90° with 1° spacing. A 30-element uniform linear array (ULA) spaced in half-wavelength units is used, and the signal-to-noise ratio (SNR) is set to be 20 dB. Here we only consider one snapshot case, and set *H* = 1, *L* = *S* = 8, *Q* = 10. Figure 4 depicts the solutions solved by different methods, and the vertical dashed lines mark the true directions. Obviously, our methods find the positions and the amplitudes exactly and achieve much sparser solutions than MP and BP. Moreover, they have a better amplitude estimation than H*l*1-DCD [40].

We give a time cost for the aforementioned methods in Table 3. From the results, we can see that our methods have the same time cost as the H*l*_1_-DCD. Although they are a little longer than MP, but they are much less costly than BP. However, the significant advantages of our methods are that they are very easy to implement in a hardware platform because only bit-shifts are needed instead of multiplications similar as [37–40], besides, they can achieve the similar reconstruction performance as BP without off-grid problem.

Next we compare the mean squared error (MSE) of position and amplitude estimation performances by applying the proposed methods and several popular methods (MP, BP, FOCUSS, SBL, H*l*1-DCD, OSR), which are defined as follows:

(20) $$\text{Position}\_\text{MSE}=\frac{{\left\|\text{Position}\left(\hat{x}\right)-\text{Position}\left(x\right)\right\|}_{2}^{2}}{{\left\|\text{Position}\left(x\right)\right\|}_{2}^{2}}$$

(21) $$\text{Amplitude}\_\text{MSE}=\frac{{\left\|\hat{x}-x\right\|}_{2}^{2}}{{\left\|x\right\|}_{2}^{2}}$$

where **x̂** is an estimate of **x**.

**Figure 5.**
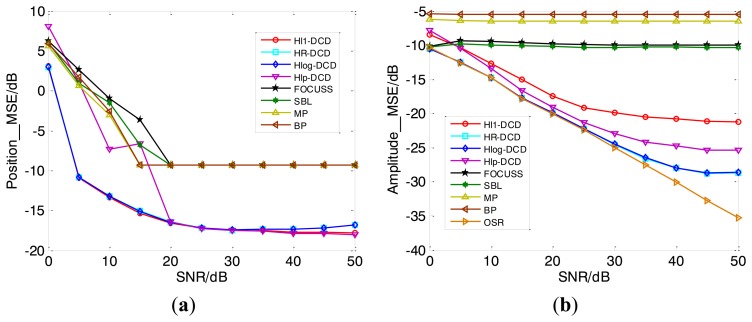
MSE of position *vs.* SNR (**a**) and amplitude *vs.* SNR (**b**). Uncorrelated source signals come from 47.7°, −28.5°, −9.2°, 11.6°, and 30.1°. Herein one snapshot is used.

Figure 5a compares the MSEs of DOA estimation results of different methods under varying SNR, which are averaged by 100 Monte Carlo trials. From the curves of MSE of position *versus* SNR shown in Figure 5a, it can be seen that H*l*_1_-DCD and the proposed methods have similar results in position estimation, and outperform their counterparts. However, our methods have better amplitude estimation accuracy than H*l*_1_-DCD from Figure 5b, and are even very close to the OSR performance.

**Figure 6.**
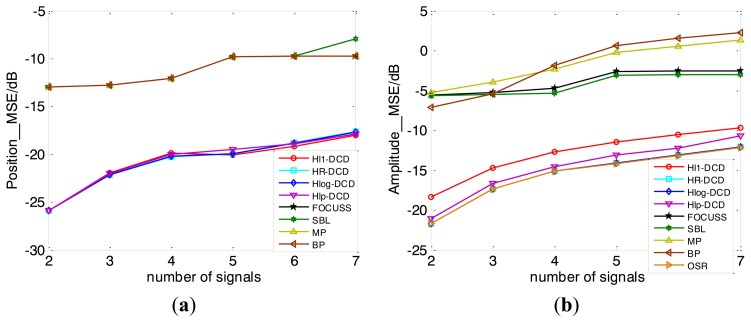
MSE of position *vs.* number of signals (**a**) and amplitude *vs.* number of signals (**b**). SNR is fixed at 20 dB.

The MSEs of DOA estimation *versus* number of signals shown in Figure 6 are obtained over 100 Monte Carlo trials with SNR equals to 20 dB. We can clearly see that our methods perform best compared to other methods with the same parameters, especially on the amplitude estimation results.

### 4.2. Passive Radar Imaging

**Figure 7.**
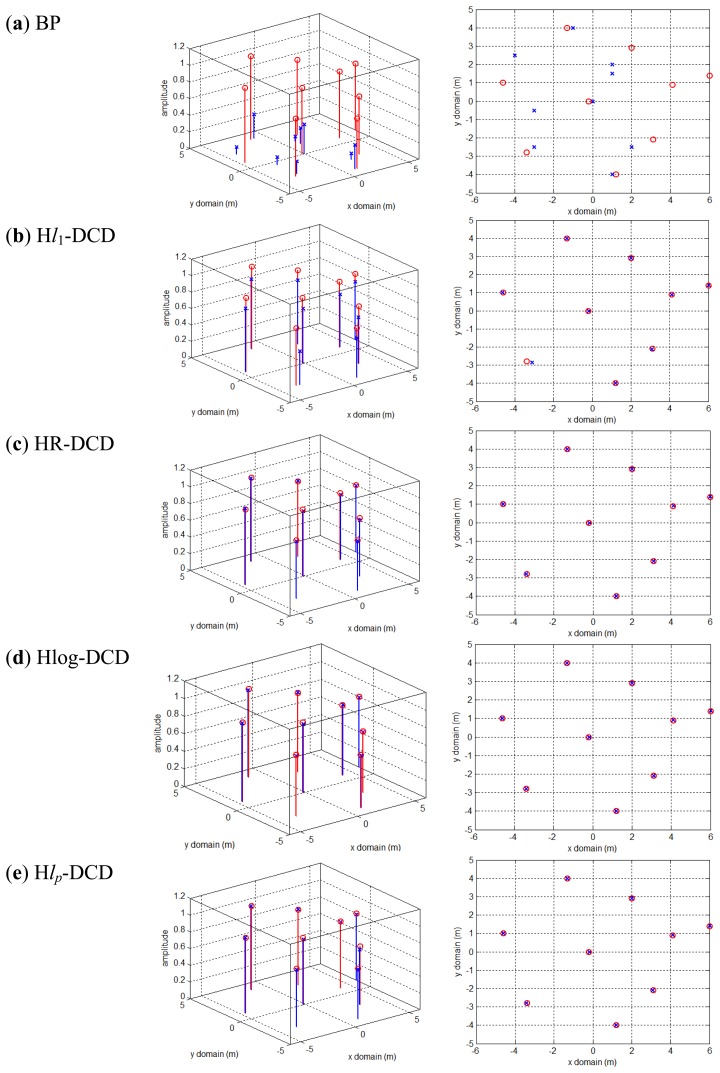
Passive radar imaging results by applying BP (**a**), H*l*_1_-DCD (**b**), HR-DCD (**c**), Hlog-DCD (**d**), H*l_p_*-DCD (**e**). Left and Right figures show the amplitude and position estimates, respectively. Red circles denote the true scatterers of the target, and blue crosses represent the estimated results.

In this part, we aim to test the performance of the proposed methods in a passive radar imaging system. Based on the final echo Equation (17) stated in [6] which is similar to Equation (2) in this paper, sparse recovery techniques can be utilized to solve the problem of passive radar imaging. In the simulation, we choose seven digital video broadcasting (DVB) satellites as opportunity transmitters and 10 receivers, and the initialization grids are 21 × 41 represented for the imaging scene 20 m × 20 m (here we use the axis function in matlab to make the scene limited to be 12 m × 10 m so as to achieve a better visual effect). There are nine off-grid scatterers with different reflection coefficients in the scene of interest as the red circles illustrated in Figure 7. The number of frequency samplings corresponding to each transmitter is 10, and the SNR equals to 20 dB. Other simulation parameters are the same as displayed in [6]. Here we set *H* = 2, *L* = *S* = 10 and grid refinement times *Q* = 10.

Figure 7a shows the poor imaging result by BP due to the reason that in practice the scatterers are not located exactly on the pre-discretized grids, and the echoes are mismatched with the measurement matrix, therefore the performances of CS-based reconstructions are generally unsatisfactory. Figure 7b is the imaging result by H*l*_1_-DCD, and we can see that it can seek the exact locations of the target, however the amplitude estimation of the reflection coefficients is not precise.

Figure 7c–e represents the imaging results by the proposed methods. As expected, the proposed methods do not have the off-grid problem from the perspective of both the position and amplitude estimation results, which show the potential of our methods to be applied in practical passive radar system.

**Figure 8.**
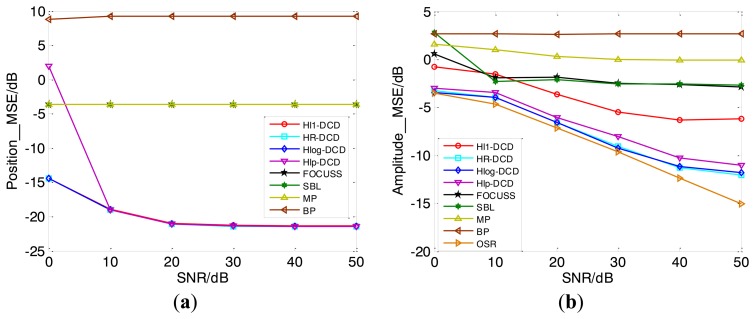
MSE of imaging results *versus* SNR. MSE of position *vs.* SNR (**a**) and amplitude *vs.* SNR (**b**).

Figure 8 shows the MSEs of position and amplitude estimation results *versus* SNR by applying different methods, which are averaged over 100 Monte Carlo trials. In the simulation, we have assumed that there are nine off-grid scatterers with unit reflection coefficients distributed randomly in imaging region. It is seen that our methods outperform the conventional methods (MP, BP, FOCUSS, SBL) in further improvement of MSEs of position and amplitude estimates when off-grid target emerges. In the meantime, they have better amplitude estimation result than H*l*_1_-DCD with the same parameters shown in Figure 8b. Moreover, the performances of our methods approach the OSR very closely under varying SNR, both in position and amplitude estimates.

### 4.3. ISAR Imaging

We utilize the CS ISAR imaging model as discussed in [43], and assume that the translational motion compensation has been already achieved and thus only the rotational motion is considered. The relation between the received signal and the complex reflection coefficients can be written as Equation (10) in [43], which is equivalent to Equation (2) in this paper.

We use the quasi real data of an airplane (B-727) provided by the U.S. Naval Research Laboratory to test the feasibility and performance of the proposed methods, which is available on the website http://airborne.nrl.navy.mil/∼vchen/tftsa.html. The stepped frequency radar operates at 9 GHz with the equivalent pulse repetition interval (PRI) of 3.2 ms and has a bandwidth of 150 MHz. For each pulse train, 64 complex range samples were saved, and the file contains 200 successive pulse trains within the long coherent processing interval (CPI). An additive noise is added to the original B-727s data, and the SNR is set to 10 dB. Herein we choose 40 range cell numbers and 20 cross range cell numbers for short CPI test, and the reconstruction of target spatial domain is discretized with 64 range bins and 32 cross range bins, besides, we set *H* = 2, *L* = *S* = 60 and *Q* = 5. Since in radar imaging field, the position estimates are very important when off-grid problem exists.

**Figure 9.**
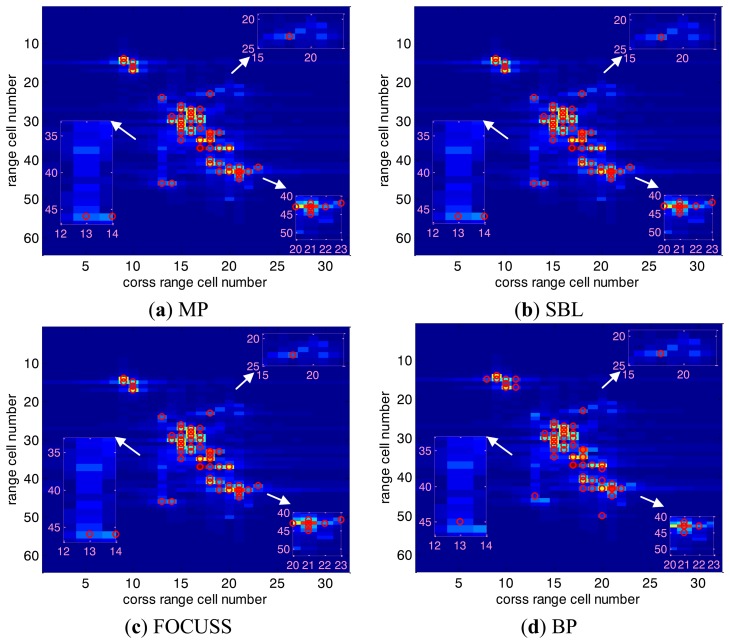

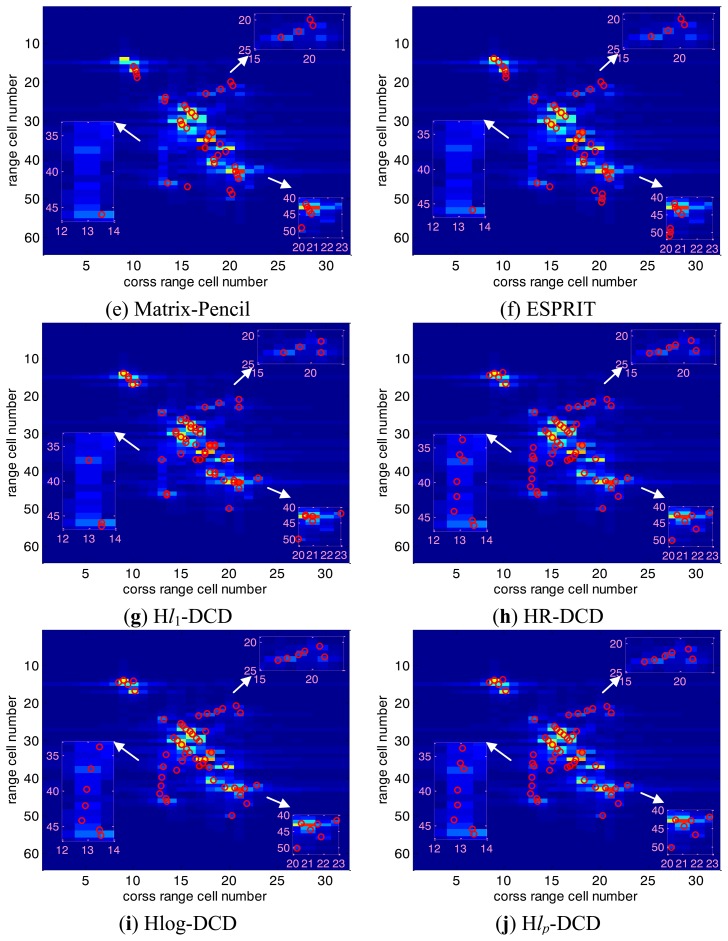
ISAR imaging results of B-727 by MP (**a**), SBL(**b**), FOCUSS (**c**), BP (**d**), Matrix-Pencil (**e**), ESPRIT (**f**), H*l*_1_-DCD (**g**), HR-DCD (**h**), Hlog-DCD (**i**), H*l_p_*-DCD (**j**). The red circles denote the corresponding position recovery result, and background represents the FFT-based reconstruction.

Figure 9 are the position recovery results by MP, SBL, FOCUSS, BP, Matrix-Pencil, ESPRIT, H*l*_1_-DCD and the proposed methods (HR-DCD, Hlog-DCD and H*l_p_*-DCD) shown as the red circles, and we use the conventional FFT-based ISAR image as the background for fair comparison. As stated above, the imaging performances of the classical methods (MP, BP, FOCUSS, SBL) are not good because of the off-grid errors. Matrix Pencil and ESPRIT can direct solve the positions of the plane, but their performances suffer from low SNR and few snapshots, therefore the imaging results are not good enough. In contrast, our methods can deal with the off-grid target, and achieve satisfying imaging performances which have better visual effect for the airplane shape. Moreover, it is obvious that the imaging results of our methods are better than H*l*_1_-DCD with the same parameters, especially on the details of airplane wings and tails as illustrated in zoomed-in regions of Figure 9h–j, which demonstrate the advantage of homotopy DCD method with non-convex regularization compared with convex regularization under the same measurements.

In order to quantitatively evaluate the amplitude estimation performances of the obtained ISAR images via different methods, we use the correlation value [44] to evaluate the similarity between the recovered images and the reference image, and the image entropy [44] to measure the focusing quality of the recovered images. They are defined as follows:

(22) $$\text{Cor}\left(x_{r},x_{o}\right)=\frac{\left|\langle x_{r},x_{o}\rangle \right|}{{\left\|x_{r}\right\|}_{2}{\left\|x_{o}\right\|}_{2}}$$

(23) $$\text{Ent}\left(x_{o}\right)=-\sum_{i}p_{i}\log p_{i}$$

where **x***_r_* and **x***_o_* denote the reference image vector using full measurements and the recovered image vector by different methods, and *p_i_* is the histogram of the recovered gray level (0∼255) image.

The correlation and entropy values of the recovered ISAR images under different methods are summarized in Table 4. It can be seen that the proposed methods have a higher correction with the reference image and a lower entropy than their counterparts, and thereby exhibit a better capability of target-information extraction.

## 5. Conclusions

Two major problems of applying CS to sparse continuous signal reconstruction are discussed in this paper, and we have presented computationally efficient and accurate methods to overcome the difficulties. For solving the off-grid problem, we utilized the grid refinement technique. In the meantime, in order to obtain high recovery performance and keep low calculation complexity, we propose a fast and accurate homotopy DCD reconstruction combined with three typical non-convex regularizations, which promotes sparsity more strongly than any convex penalty function can. Extensive experiments have been conducted to validate and compare the performances of the proposed methods with several popular solvers. Our future work will try to synthesize with parallel sparse optimization technique using multi-core CPUs/GPUs [45], which may provide a viable solution to real-time potential applications.

## Figures and Tables

**Table 1.** DCD Algorithm.

**Step**	**Equation**

	**Initialization: x = 0, r = y, R = A***^H^***A**, *m* = 0, *δ* = *H*
1	for *m =* 1 to *M*_b_, repeat:
2	*δ* = *δ*/2, *α* = [*δ*,− *δ*, *jδ*, − *jδ*]
3	for *i =* 1 to *N*, repeat:
4	for *k* = 1 to 4, repeat:
5	Δ*J* = *δ*^2^*R_i_*,_i_/2 − Re{ $\alpha_{k}^{*}$ (**A**^(^*^i^*^)^)*^H^* **r**}+τ(*φ*(*x_i_*+*α_k_*) − *φ*(*x_i_*))
6	if Δ*J* < 0, do:
7	*x_i_* ← *x_i_* + *α_k_*, **r** =**r** − *α_k_***A**^(^*^i^*^)^
8	end if
9	end for
10	end for
11	end for

**Table 2.** HR-DCD Algorithm.

**Step**	**Equation**

	**Initialization: x = 0, *I* = Ø, r = y, b = A***^H^* **r, R = A***^H^* **A**
1	Choose the first (*t*-th) element into the support according to:
	*t* = arg max*_k_* |*b_k_*|^2^; *I* ={*t*}, *τ* = |*b_t_*|
2	Repeat until a termination condition is met:
3	Solve $\min_{x_{I}}{\left\|y-A_{I}x_{I}\right\|}_{2}^{2}/2+\tau \sum_{n\in I}\left|x_{n}\right|/\left(1+a\left|x_{n}\right|/2\right)$ on the support *I* and update **r** using DCD iterations
4	Update the regularization parameter: *τ* ← *γτ*, 0 < *γ* < 1
5	Remove the *t*-th element from *I* according to the rule:
	t=argmink∈I|xk|2Rk,k/2+Re{xk*bk}−τ|xk|/(1+a|xk|/2)s.t.|xt|2Rt,t/2+Re{xt*bt}−τ|xt|/(1+a|xt|/2)<0
	If the *t*-th element is removed, update **r: r ← r +** *x_t_***A**^(^*^t^*^)^, *I* ← *I*/*t*
6	Add the *t*-th element into *I* according to the rule:
	$\begin{array}{cc} t=\arg\max_{k\in I^{c}}\left(a\left|b_{k}\right|-R_{k,k}\right)/aR_{k,k}^{2}\cdot \left\{{\left(R_{k,k}+a\left|b_{k}\right|\right)}^{2}-4aR_{k,k}\tau \right\} & s.t.{\left(R_{t,t}+a\left|b_{t}\right|\right)}^{2} \end{array}>4aR_{t,t}\tau$
	If the *t*-th element is added, update: *I* ← *I* ∪ *t*

**Table 3.** Time consumption of different methods.

	**MP**	**BP**	**H*l*_1_-DCD**	**HR-DCD**	**Hlog-DCD**	**H*l****_p_***-DCD**
Time(s)	0.013	0.721	0.130	0.122	0.129	0.126

**Table 4.** Correlation and entropy values by different methods.

	**MP**	**SBL**	**FOCUSS**	**BP**	**Matrix Pencil**	**ESPRIT**	**H*l*_1_-DCD**	**HR-DCD**	**Hlog-DCD**	**H*l****_p_***-DCD**
***Cor***	0.817	0.890	0.887	0.913	0.816	0.798	0.925	0.952	0.957	0.965
***Ent***	0.826	0.811	0.836	0.859	0.810	0.802	0.713	0.689	0.683	0.613

## Appendix

***Remark 1*:** *the Penalty is Logarithmic Function*.

***Proof for Proposition 3***: Let the *t*-th element *x_t_* = 0 be activated as *x̂_t_* = *α* = |*α*|*e ^j^*^arg{^*^α^*^}^, and the updated solution vector is denoted as **x̂**. The update of the cost function is then given by

(24) $$\Delta J=J\left[\hat{x}\right]-J\left[x\right]=\frac{1}{2}{\left|\alpha \right|}^{2}R_{t,t}-ℜ\left\{\alpha^{*}b_{t}\right\}+\tau \frac{1}{a}\log\left(1+a\left|\alpha \right|\right)$$

The cost function is reduced if Δ*J* < 0. For a fixed |*α*|, Δ*J* achieves a minimum if arg{*α*} = arg{*b_t_*}, and in this case

(25) $$\Delta J=\frac{1}{2}{\left|\alpha \right|}^{2}R_{t,t}-\left|\alpha \right|\left|b_{t}\right|+\tau \frac{1}{a}\log\left(1+a\left|\alpha \right|\right)$$

As we can see, if |*α*| = 0, then Δ*J* = 0. So if ${\frac{\partial \Delta J}{\partial \left|\alpha \right|}|}_{\left|\alpha \right|=0}<0$, when|*α*| > 0, there exists a |*α*| that makes Δ*J* < 0.

(26) $$\frac{\partial \Delta J}{\partial \left|\alpha \right|}=R_{t,t}\left|\alpha \right|-\left|b_{t}\right|+\frac{\tau }{1+a\left|\alpha \right|}$$

If ${\frac{\partial \Delta J}{\partial \left|\alpha \right|}|}_{\left|\alpha \right|=0}<0$, we can get

(27) $$\tau -\left|b_{t}\right|<0$$

Therefore, if |*b_t_*| > *τ*, there exists a |*α*| that decreases the cost function.

***Proof for Proposition 4***: Let the *t*-th element *x_t_* ≠ 0 be activated as *x̂_t_* = 0, and the updated solution vector is denoted as **x̂**. The update of the cost function is then given by

(28) $$\Delta J=J\left[\hat{x}\right]-J\left[x\right]=\frac{1}{2}{\left|x_{t}\right|}^{2}R_{t,t}+ℜ\left\{x_{t}^{*}b_{t}\right\}-\tau \frac{1}{a}\log\left(1+a\left|x_{t}\right|\right)$$

Thus, if 12|xt|2Rt,t+Re{xt*bt}−τ1alog(1+a|xt|)<0, then Δ*J* < 0, there exists a nonzero value of the *t*-th element that decreases the cost function that should be removed from the support.

**Remark 2**: The Penalty is l_p_ Norm Function.

***Proof for Proposition 5***: Let the *t*-th element *x_t_* = 0 be activated as *x̂_t_* = *α* = |*α*|*e ^j^*^arg{^*^α^*^}^, and the updated solution vector is denoted as **x̂**. The update of the cost function is then given by

(29) $$\Delta J=J\left[\hat{\text{x}}\right]-J\left[x\right]=\frac{1}{2}{\left|\alpha \right|}^{2}R_{t,t}-ℜ\left\{\alpha^{*}b_{t}\right\}+\tau {\left|\alpha \right|}^{p}$$

The cost function is reduced if Δ*J* < 0. For a fixed |*α*|, Δ*J* achieves a minimum if arg{*α*} = arg {*b_t_*}, and in this case

(30) $$\Delta J=\frac{1}{2}{\left|\alpha \right|}^{2}R_{t,t}-\left|\alpha \right|\left|b_{t}\right|+\tau {\left|\alpha \right|}^{p}=\frac{1}{2}R_{t,t}{\left(\left|\alpha \right|-\frac{\left|b_{t}\right|}{R_{t,t}}\right)}^{2}+\tau {\left|\alpha \right|}^{p}-\frac{{\left|b_{t}\right|}^{2}}{2R_{t,t}}$$

From Equation (30) we can see that if $\left|\alpha \right|=\frac{\left|b_{t}\right|}{R_{t,t}}$ and $\tau {\left|\alpha \right|}^{p}-\frac{{\left|b_{t}\right|}^{2}}{2R_{t,t}}<0$, then Δ*J* < 0.

Thus, the value of |*α*| results in the decrement of the cost function is given by

(31) $$\alpha =\frac{\left|b_{t}\right|}{R_{t,t}}e^{j\arg\left\{b_{t}\right\}}$$

And we can get the proposition that if ${\left|b_{t}\right|}^{2-p}>2\tau R_{t,t}^{1-p}$, the *t*-th element would be added into the support.

***Proof for Proposition 6***: Let the *t*-th element *x_t_* ≠ 0 be activated as *x̂_t_* = 0, and the updated solution vector is denoted as **x̂**. The update of the cost function is then given by

(32) $$\Delta J=J\left[\hat{x}\right]-J\left[x\right]=\frac{1}{2}{\left|x_{t}\right|}^{2}R_{t,t}+ℜ\left\{x_{t}^{*}b_{t}\right\}-\tau {\left|x_{t}\right|}^{p}$$

Thus, if 12|xt|2Rt,t+Re{xt*bt}−τ|xt|p<0, then Δ*J* < 0, there exists a nonzero value of the *t*-th element that decreases the cost function that should be removed from the support.
